# Real-World Hypoglycemia Avoidance with a Continuous Glucose Monitoring System's Predictive Low Glucose Alert

**DOI:** 10.1089/dia.2018.0359

**Published:** 2019-03-30

**Authors:** Sarah Puhr, Mark Derdzinski, John B. Welsh, Andrew Scott Parker, Tomas Walker, David A. Price

**Affiliations:** Dexcom, Inc., San Diego, California.

**Keywords:** Alarms, Alerts, Continuous glucose monitoring, Hypoglycemia, Predictive alerts

## Abstract

***Background:*** Programmable and fixed auditory and/or vibratory threshold alerts are essential features of real-time continuous glucose monitoring (rtCGM) systems that provide users time to intervene before the onset of clinical hypoglycemia or hyperglycemia. A sixth-generation rtCGM system from Dexcom, Inc. (G6) includes a new alert that is triggered when an algorithm predicts that an estimated glucose value ≤55 mg/dL will occur within 20 min, allowing users more time to act to avoid hypoglycemia. We examined whether this predictive low glucose alert provided added benefit to traditional low threshold alerts.

***Methods:*** We analyzed glucose values from an anonymized sample of 1424 patients who transitioned to G6 from the preceding fifth-generation system (G5) with no predictive alert. Users with the low threshold alert setting of 70 or 80 mg/dL were evaluated separately. Receiver users, those who disabled the predictive low glucose alert, or those with <30 days of data immediately before or after the transition to G6 were excluded.

***Results:*** Percent time <54, ≤55, <70, and >250 mg/dL fell significantly after the transition to G6, independent of low threshold alert setting. Time in range improved for G6 users with a low threshold alert setting of 70 mg/dL.

***Conclusions:*** Advance warning provided by predictive low glucose alerts may further reduce hypoglycemia among rtCGM-experienced users.

## Introduction

Real-time Continuous Glucose Monitoring (rtCGM) systems are important diabetes management tools that transmit glucose measurements at regular intervals from a wearable body sensor to a nearby receiver or mobile device through Bluetooth, providing users with actionable information on historic and current glucose concentration and velocity of glucose change. Currently available rtCGM systems offer programmable and fixed auditory and/or vibratory alerts that are activated in response to existing or impending hypoglycemia or hyperglycemia or to rapidly changing glucose concentrations. RtCGM systems differ from the currently available intermittently scanned continuous glucose monitoring (isCGM) system, which provides historic and current glucose data and velocity of glucose change only upon active scanning of a wearable body sensor by a handheld reader. Unlike rtCGM systems, the FDA-approved version of the isCGM system does not provide auditory or vibratory alerts or alarms.^1^

Randomized controlled clinical trials that examined the efficacy of modern rtCGM in participants with type 1 diabetes (T1D) treated with multiple daily injections (MDI), such as the DIAMOND^2–5^ and HypoDE trials,^6^ enabled device alerts and alarms. Given the individual variability in hypoglycemia awareness and risk for hypoglycemia, participant threshold alert settings were personalized by clinicians for these trials. Median (interquartile range) time in measured hypoglycemia at study end was reduced to 4 (0–13) min <50 mg/dL daily in DIAMOND^2^ and 3.8 (1.1–11.9) min <54 mg/dL daily in HypoDE.^6^ In contrast, IMPACT studied the efficacy of isCGM—absent alerts and alarms—in participants with T1D treated with MDI and demonstrated that mean (standard deviation) time in measured hypoglycemia was 48.0 (57.6) min daily <55 mg/dL, 18.6 (25.8) min of which occurred at night.^7^ This difference in time spent in hypoglycemia between rtCGM and isCGM system use supports the premise that low threshold alerts are important components of rtCGM systems.

The goal of low threshold alerts is to warn patients and their caregivers of hypoglycemic events before neuroglycopenia develops. Although the glucose threshold at which cognitive impairment is first detected or observed may vary between individuals, neuroglycopenic symptoms commonly occur at or <55 mg/dL.^8^ Consensus statements have recommended that low threshold alerts be set at 70 mg/dL to provide patients enough time to intervene before neuroglycopenia/clinical hypoglycemia (i.e., glucose <55 mg/dL).^9^ Further research demonstrated that a higher low threshold alert setting of 80 mg/dL provided additional time for interventions before the onset of clinical hypoglycemia compared with a low threshold alert setting of 70 mg/dL^10^; more advance warning may be particularly beneficial to those with impaired awareness of hypoglycemia or a history of problematic hypoglycemia. Davey et al. showed that short-term rtCGM use with a low threshold alert enabled at 80 mg/dL reduced the incidence of hypoglycemia ≤65 mg/dL by 44% in patients with T1D compared with rtCGM use with alerts disabled.^11^ Analysis of real-world rtCGM data supported these findings and concluded that pediatric and adult patients with a low threshold alert set at ≥80 mg/dL had a higher mean glucose than those with a low threshold alert set at <80 mg/dL.^12,13^ However, higher low threshold alert settings are only practical in more accurate rtCGM systems, where sensitivity and specificity are high; inaccurate “nuisance” alerts have been cited as a primary reason for rtCGM therapy discontinuation.^14–17^ The incorporation of a predictive low glucose alert into a clinically accurate rtCGM system may allow a user to set a lower low threshold alert, while decreasing nuisance alerts at “normal” glucose levels and minimizing clinical hypoglycemia. Earlier *in silico* studies support this hypothesis.^18,19^

We examined whether a predictive low glucose alert could provide additional advance warning to rtCGM users before the onset of clinical hypoglycemia by evaluating estimated glucose values (EGVs) from an anonymized convenience sample of 1424 patients before and after their transition from an earlier fifth-generation (G5) system with no predictive alert to a comparably accurate^20,21^ sixth-generation (G6) system with predictive “Urgent Low Soon” (ULS) alert. The ULS alert is enabled by default and triggered when an EGV ≤55 mg/dL is predicted in the next 20 minutes; the activation of a ULS alert inhibits low threshold alert activations in the following 30 minutes to limit alarm fatigue.

## Methods

We examined EGVs from an anonymized convenience sample of patients who used the G5 mobile and transitioned to the G6 (both from Dexcom, Inc., San Diego, CA) between May 1, 2018, and August 31, 2018. Patients were included if they had uploaded one or more EGVs during each of the 30 days of data immediately before and after the transition to G6. Only patients using Internet-connected mobile devices, which passively and continuously upload data to the Dexcom Cloud, were included. Users with a low threshold alert setting of 70 or 80 mg/dL (default) were included. Data from patients who disabled the ULS alert on their G6 systems, or those from patients who varied their low threshold alert setting at any time during the study interval, were excluded. A total of 1424 patients met the selection criteria.

Data from users with a low threshold alert setting of 70 mg/dL (*n* = 658) or 80 mg/dL (*n* = 766) were evaluated separately. Hypoglycemia exposure, hyperglycemia exposure, and time in range (TIR) were calculated as the percentage of EGVs below, above, and within certain thresholds, respectively. Hypoglycemia exposure was evaluated as the percentage of EGVs ≤55 mg/dL, to match the ULS alert setting, as well as the percentage of EGVs <54 mg/dL, to match consensus guidelines.^22^ *P*-values were computed using a two-sided Welch's unequal variance *t*-test between population means.

## Results

The ULS alert remained enabled among >97% of G6 users (not shown) and was triggered less than once daily on average (Table 1). Users who had a low threshold alert set at 80 mg/dL trended toward having fewer ULS notifications daily than those with a low threshold alert set at 70 mg/dL (0.6 ± 0.6 vs. 0.9 ± 0.8 times daily, respectively).

**Table 1.** Urgent Low Soon Activations and Mean (Standard Deviation) Continuous Glucose Monitoring–Derived Glycemic Metrics of G5 Users Who Transitioned to G6 with the Urgent Low Soon Alert Enabled

	*Low threshold alert setting of 70 mg/dL (*n* = 658)*			*Low threshold alert setting of 80 mg/dL (*n* = 766)*		
	*G5*	*G6*	P*-value*	*G5*	*G6*	P*-value*
ULS activations/day	—	0.9 (0.8)	—	—	0.6 (0.6)	—
Mean glucose (mg/dL)	165.4 (33.0)	163.0 (31.4)	0.18	174.2 (30.3)	172.2 (29.8)	0.19
Time <54 mg/dL (%)	1.0 (1.5)	0.6 (1.0)	<0.001	0.6 (1.1)	0.4 (0.8)	<0.001
Time ≤55 mg/dL (%)	1.2 (1.7)	0.8 (1.2)	<0.001	0.7 (1.3)	0.5 (0.9)	<0.001
Time <70 mg/dL (%)	3.7 (3.5)	3.0 (3.0)	<0.001	2.3 (2.8)	2.0 (2.5)	0.006
TIR (70–180 mg/dL) (%)	61.1 (17.9)	63.0 (18.1)	0.05	57.5 (17.5)	58.7 (18.1)	0.18
Time >180 mg/dL (%)	35.2 (19.1)	34.0 (19.1)	0.23	40.2 (18.4)	39.4 (18.9)	0.37
Time >250 mg/dL (%)	12.6 (12.1)	11.1 (11.2)	0.02	14.4 (11.8)	13.0 (11.5)	0.02

G5, fifth-generation; G6, sixth-generation; TIR, time in range; ULS, “Urgent Low Soon”.

The transition to G6 was associated with significantly reduced biochemical (<70 mg/dL) and clinical (<54 mg/dL) hypoglycemia, independent of low threshold alert setting (Table 1). Compared with intervals of G5 use, the extent of clinical hypoglycemia fell by 40.0% and 33.3% during G6 use for users with a low threshold setting of 70 and 80 mg/dL, respectively. Exposure to biochemical and clinical hypoglycemia was slightly lower overall for users with a threshold alert setting of 80 mg/dL compared with those with a threshold alert setting of 70 mg/dL.

Mean glucose was not significantly different before and after the transition to G6, either for users with a low threshold alert setting of 70 or 80 mg/dL. Mean glucose was slightly higher overall for users with a low threshold alert setting of 80 mg/dL compared with users with a low threshold alert setting of 70 mg/dL, supporting prior studies.^12,13^ TIR improved for users who had a threshold alert setting of 70 mg/dL (*P* = 0.05), but was not significantly different before and after the transition to G6 for users who had a threshold alert setting of 80 mg/dL (*P* = 0.18). Although time spent >180 mg/dL was not significantly different, time spent >250 mg/dL fell significantly after the transition to G6 for users of both low threshold alert settings, suggesting that reduced hypoglycemia did not come at the expense of increased severe hyperglycemia.

## Discussion and Conclusion

A pilot randomized controlled study demonstrated that rtCGM but not isCGM therapy reduced exposure to hypoglycemia in patients with T1D and impaired awareness of hypoglycemia, suggesting that threshold alerts unique to rtCGM systems are important for hypoglycemia avoidance.^23,24^ The current data demonstrate that the incorporation of a predictive low glucose alert into a G6 CGM system further diminishes hypoglycemia exposure, significantly reducing hypoglycemia relative to a CGM system without the predictive alert, independent of threshold alert setting. It is not surprising that users with a threshold alert setting of 80 mg/dL tended to spend less time in hypoglycemia overall compared with those with a threshold alert setting of 70 mg/dL; having a low threshold alert of 80 mg/dL provides more advanced warning to intervene before hypoglycemia.^10^ Users with a low threshold alert of 80 mg/dL also tended toward fewer ULS notifications daily, likely because they treated impending hypoglycemia after the primary low threshold alert notification. The low rate at which the ULS alert was disabled suggests that it was well tolerated and perceived as beneficial, rather than a nuisance.

The ULS alert-driven reduction in hypoglycemia did not come at the expense of increased hyperglycemia. In fact, more severe hyperglycemia (>250 mg/dL) decreased significantly, which was unanticipated but may be attributable to prevention of symptomatic hypoglycemia and/or fewer episodes of disproportionate carbohydrate intake after ULS alert notification. That said, the methods used to treat hypoglycemia in this study—omitting or reducing insulin doses, eating, or both—are unknown. This is in contrast to low glucose suspend (LGS) or predictive low glucose suspend (PLGS) automated insulin delivery systems, which suspend insulin in response to existing or impending hypoglycemia, respectively. Benefits of LGS and PLGS systems compared with sensor-augmented pump therapy have been demonstrated.^25–28^

A strength of this study is that it tracked the same 1424 patients for 30 days before and after their transition to G6, making it unlikely that other nondevice-related behaviors were responsible for the observed reductions in hypoglycemia and severe hyperglycemia. However, other device settings, such as high threshold alerts, were not evaluated and may have contributed to the observed results. This study was limited by the narrow population of Dexcom users who were evaluated; among patients who transitioned to G6 immediately after the launch were those whose experience with CGM may not be representative of the population at-large. Moreover, excluding individuals who accessed their CGM data on a receiver alone may have biased the study population. Additional studies are needed to understand the benefit in subpopulations, such as patients with impaired awareness of hypoglycemia, pediatric patients, or patients on Medicare.
